# A case of salvage surgery following chemoradiotherapy and durvalumab for initially unresectable superior sulcus tumor with N3 involvement

**DOI:** 10.1186/s44215-024-00169-z

**Published:** 2024-10-01

**Authors:** Takehiko Manabe, Masatoshi Kanayama, Hiroki Matsumiya, Katsuma Yoshimatsu, Masataka Mori, Natsumasa Nishizawa, Akihiro Taira, Masaru Takenaka, Koji Kuroda, Koichi Azuma, Fumihiro Tanaka

**Affiliations:** 1 Second Department of Surgery, University of Occupational and Environmental Health, Kitakyushu, Japan; 2 Division of Respirology, Neurology, and Rheumatology, Department of Internal Medicine, Kurume University School of Medicine, Kurume, Fukuoka Japan

**Keywords:** Immune checkpoint inhibitor, Lung cancer, Salvage surgery, PACIFIC trial, Superior sulcus tumor

## Abstract

**Background:**

Durvalumab after chemoradiation (PACIFIC regimen) provides favorable treatment outcomes for unresectable stage III non-small cell lung cancer (NSCLC). The feasibility of salvage surgery after the PACIFIC regimen has been reported in some studies; however, its efficacy remains unclear. We herein present the first case of salvage surgery after the PACIFIC regimen for a superior sulcus tumor with N3 involvement, in which a pathological complete response was achieved.

**Case presentation:**

A 53-year-old man with a left superior sulcus tumor with N3 (# 1L, #4R) involvement (adenocarcinoma, clinical T3N3M0/IIIC) underwent concurrent chemoradiotherapy (2 cycles of cisplatin plus vinorelbine with 60 Gy radiotherapy) followed by durvalumab treatment for 1 year at a previous hospital. The PACIFIC regimen provided a significant primary tumor shrinkage (diameter 3.1 cm to 0.5 cm) with the disappearance of 18F-fluorodeoxyglucose uptake in all nodes. Six months after the end of the PACIFIC regimen, only the primary tumor showed enlargement (diameter 0.5 cm to 2.0 cm). Accordingly, local tumor recurrence was suspected. Salvage surgery (left upper lobectomy with combined chest wall resection [1st to 4th rib]) was performed. The histological examination revealed no viable tumor cells (ypT0N0M0). At 7 months after salvage surgery, the patient remains alive with no signs of tumor recurrence.

**Conclusions:**

The present case suggests that salvage surgery may be feasible after the PACIFIC regimen for superior sulcus tumors. A long-term follow-up is essential to determine the efficacy of salvage surgery.

## Background

Immunotherapy with durvalumab after concurrent chemoradiotherapy (PACIFIC regimen) has become a standard treatment of care for unresectable stage III non-small cell lung cancer (NSCLC) because of its significant survival benefits [1]. While previous studies may indicate the feasibility and efficacy of salvage surgery after immunotherapy [2], the specific role of salvage surgery after the PACIFIC regimen requires further investigation [2–5]. In this report, we present the first case of salvage surgery after the PACIFIC regimen for an initially unresectable superior sulcus tumor with N3 involvement, in which a complete pathological response was achieved.

## Case presentation

**Fig. 1:**
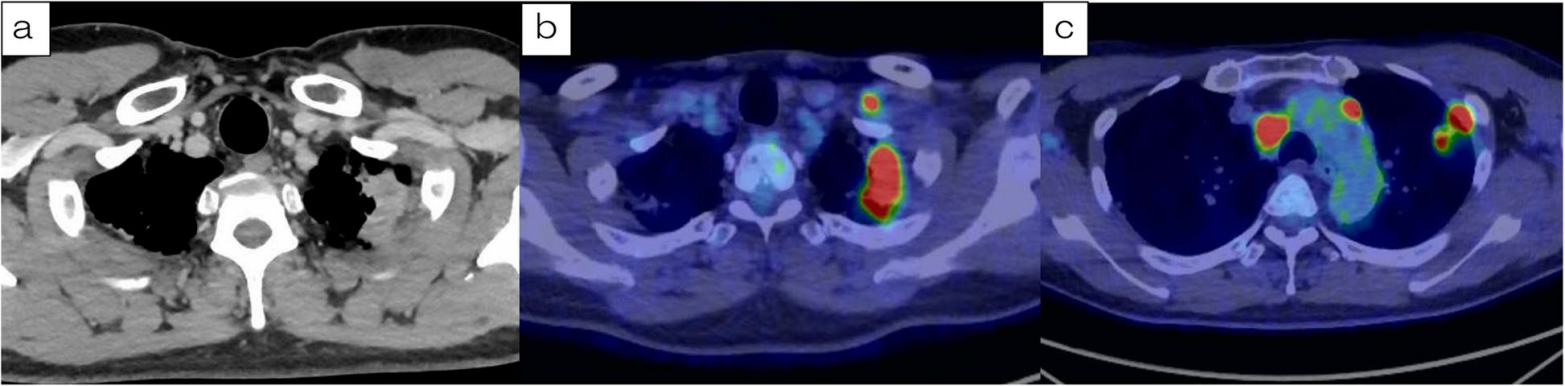
Computed tomography (CT) and positron emission tomography (PET) before chemoradiotherapy (CRT) followed by durvalumab. **a** CT at the initial diagnosis revealed a tumor of 3.1 cm in diameter originating from the left upper lobe in the left superior sulcus that was in contact with the chest wall and enlarged hilar, periaortic, and left supraclavicular lymph nodes. **b**, **c** Fluorodeoxyglucose (FDG)-PET revealed uptake in the primary lesion, left supraclavicular lymph nodes, mediastinal lymph nodes, and periaortic lymph nodes. The maximum standardized uptake value on FDG-PET in the primary lesion was 14.1

**Fig. 2:**
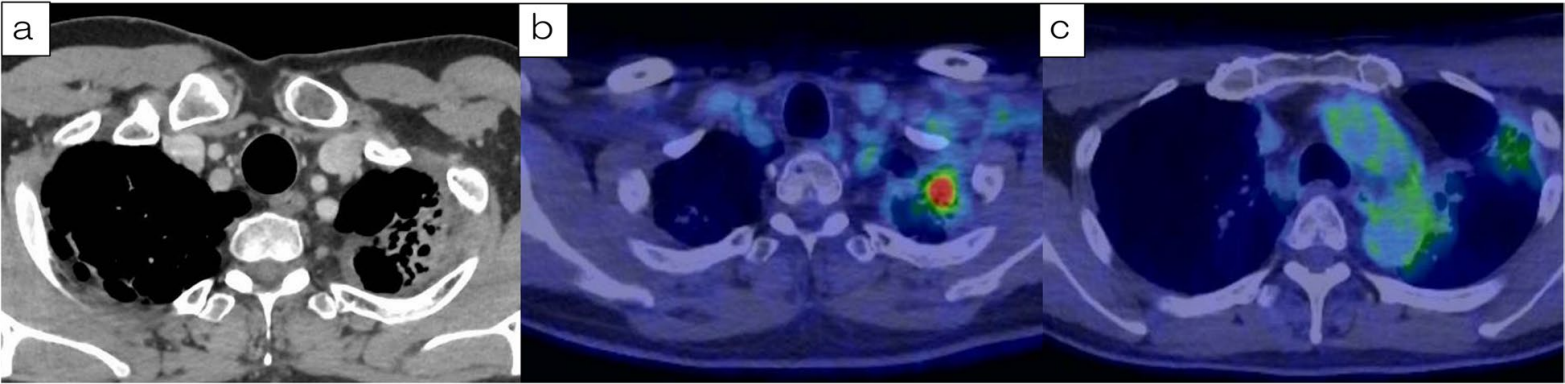
**a** The primary lesion showed a reduction in size. **b** The uptake of FDG in the primary lesion persisted. **c** The abnormal FDG uptake of FDG in the lymph nodes disappeared, and the treatment response was classified as a partial response (PR) according to the RECIST criteria

**Fig. 3:**
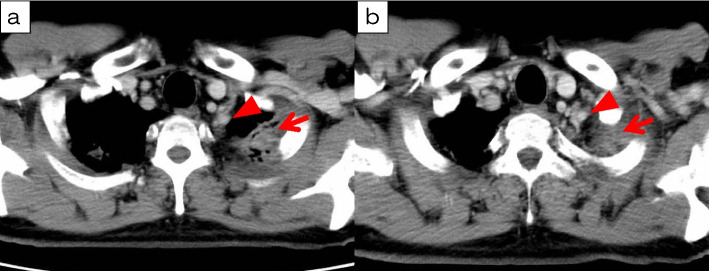
**a** During follow-up, the solid portion of the primary lesion (arrow) has enlarged, with an observed increase in contrast enhancement, raising suspicion of a residual tumor. The arrowhead indicates the left subclavian artery. **b** Regarding its relationship with the left subclavian artery (arrowhead), the tumor border (arrow) was well-defined. Preoperatively, it was predicted that there would be no direct infiltration

A 53-year-old man was admitted to our hospital for salvage surgery. Twenty months prior to his admission, he was diagnosed with a left superior sulcus tumor with N3 (#1L, #4R) involvement (NSCLC, clinical stage IIIC/T3N3M0), originating from the left side. Chest computed tomography (CT) revealed a 3.1-cm tumor with significant contrast enhancement and bronchodilation (Fig. 1). A pathological diagnosis of adenocarcinoma (no driver-gene alteration; proportion of tumor expressing programmed death-ligand 1 [PD-L1], 30%) with mediastinal and contra-mediastinal lymph node involvement was made based on a transbronchial tumor biopsy. Thereafter, the patient received concurrent chemoradiotherapy (CRT) consisting of 2 cycles of cisplatin plus vinorelbine combined with radiotherapy (60 Gy in 30 fractions). The radiation field included the primary lesion, lymph nodes #1L and #4R, as well as the hilar and mediastinal lymph nodes. After this concurrent CRT, the primary lesion was reduced in size from 3.1 to 2.7 cm. Additionally, lymph node #1L shrunk from 1.6 to 0.2 cm, and lymph node #4R reduced from 1.8 to 0.3 cm. These changes were assessed as a partial response (PR) according to the RECIST criteria. Then, consolidation treatment with durvalumab (10 mg/kg, every 2 weeks for 1 year) was performed, which provided a dramatic radiographic response (Fig. 2) with a 70% decrease in the sum of the tumor diameters (before PACIFIC regimen vs. after PACIFIC regimen: primary tumor, 3.1 cm vs. 0.5 cm; lymph nodes, 3.5 cm vs. 0.8 cm), which was equivalent to a PR. The accumulation of fluorodeoxyglucose (FDG) in the initially enlarged mediastinal and supraclavicular lymph nodes was significantly reduced and was only observed in the primary lesion. Six months later, follow-up CT showed regrowth of the primary lesion (0.5 to 2.0 cm) while the size of all lymph nodes remained stable (Fig. 3). The maximum standardized uptake value of the primary lesion on 18F-fluorodeoxyglucose-positron emission tomography (FDG-PET) was 7.7. Whole-body CT and FDG-PET showed no signs of nodal or distant metastasis, leading to a diagnosis of local tumor recurrence and potentially resectable disease. The patient was subsequently referred to our hospital for surgery.

**Fig. 4:**
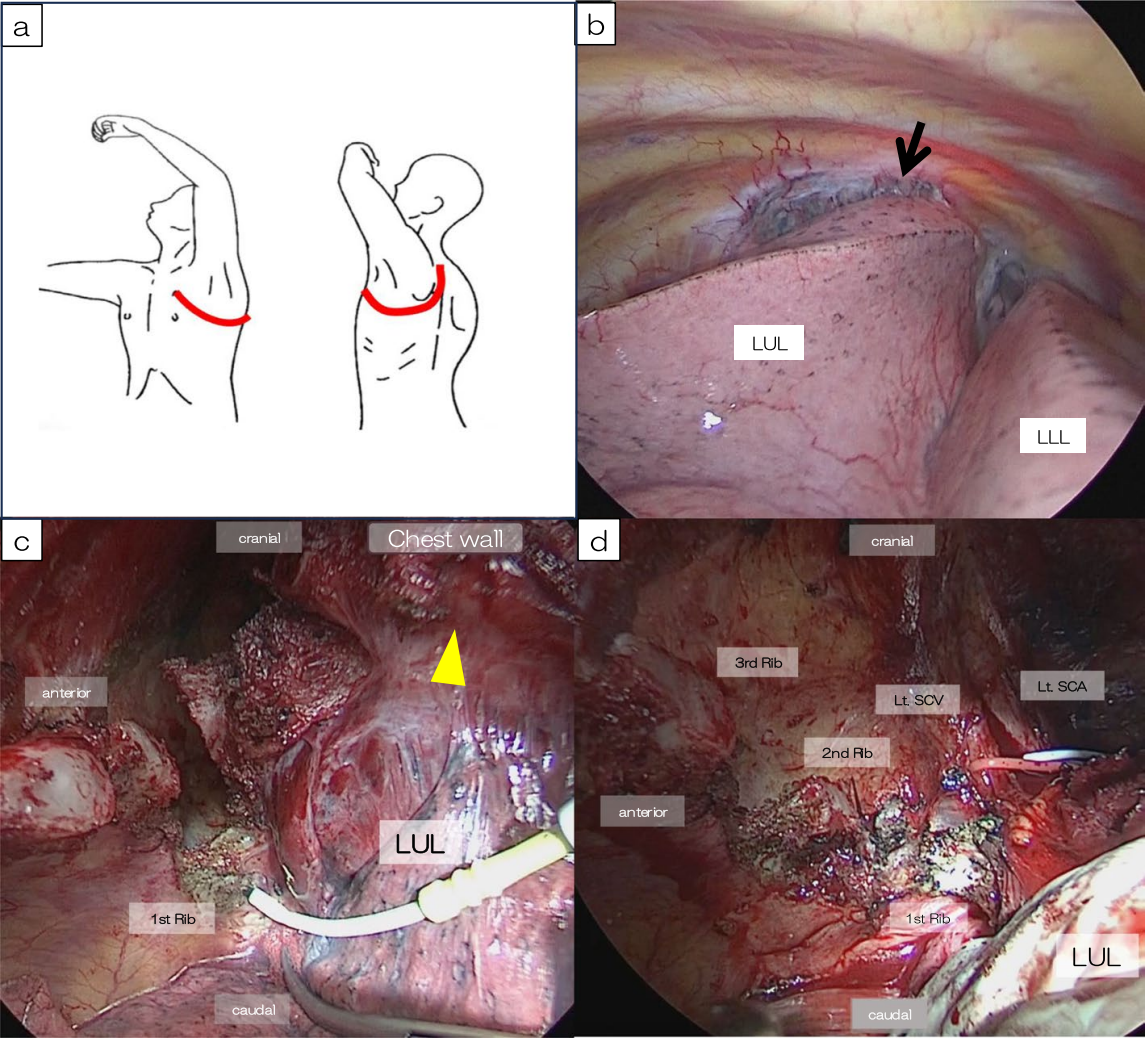
Intraoperative findings. **a** Thoracotomy incision: an incision was made posterior-laterally through the 4th intercostal space and extended anteriorly and upward, following the “Hook approach.” **b** Significant adhesions (arrow) were observed at the apical part of the left upper lobe. **c** Adhesions (arrowhead) between the left upper lobe (LUL) and the chest wall were too strong to separate, so we proceeded with the combined resection of the chest wall from the 1st to 4th ribs. LLL: left lower lobe. **d** Before performing a combined resection of the chest wall, we safely taped the left subclavian artery (Lt. SCA), vein (Lt. SCV), and brachial plexus

Through a posterolateral thoracotomy and extending the incision anteriorly at the fourth intercostal space (Fig. 4a), a left upper lobectomy with combined resection of the chest wall (1st to 4th ribs) and standard nodal dissection was performed. During the surgery, severe adhesions were identified around the apical area of the left lung, where residual FDG accumulation had been observed preoperatively (Fig. 4b). To address this, first, the left subclavian vein and brachial plexus were taped, and then chest wall resection was performed in the vicinity of the 1st to 4th ribs, where the adhesions were present (Fig. 4c, d). The bronchial ends were anastomosed end-to-end with interrupted 4–0 polydioxanone (PDS, Ethicon Inc., Somerville, NJ, USA) sutures and were covered with detached pericardial fat. The chest wall was reinforced with mesh. Intraoperative frozen sections of the main lesion and dissected lymph nodes showed no malignant cells.

A pathological examination revealed no viable tumor cells in any resected specimen, including the lymph nodes. Bleeding, hemosiderin deposition, and infiltration of atypical multinucleated giant cells were found in the resected lung, indicating alterations that could have been induced by preoperative treatment. The inflammation observed here was considered to be the cause of the uptake observed on preoperative FDG-PET. Seven days after the surgery, a CT scan revealed a pulmonary embolism in the left peripheral pulmonary artery (common terminology criteria for adverse events [CTCAE] version 5.0; Grade 1). The patient was treated with apixaban at a dose of 20 mg/day. After the initiation of this medication, CT showed a reduction in the embolism, and the patient was discharged 24 days after surgery. At 7 months after surgery, the patient was alive with no signs of tumor recurrence.

## Discussion

The efficacy of immunotherapy in neoadjuvant or perioperative settings for patients with resectable NSCLC has been demonstrated in some clinical trials, including CheckMate 816 [6–8]. Furthermore, in the PACIFIC trial, treatment with durvalumab after CRT for unresectable stage III NSCLC has shown favorable outcomes; based on five-year follow-up data, the estimated overall survival (OS) and progression-free survival (PFS) rates are reported to be 42.8% and 33.3%, respectively [1].

Since the clinical introduction of the PACIFIC regimen, some reports have discussed salvage surgery following this treatment [2, 3, 5, 9–11]. Despite the potential advantages of superior anti-tumor effects, safety concerns exist regarding surgery following the PACIFIC regimen. To the best of our knowledge, there have been 15 reported cases of salvage surgery after this regimen (Table 1). In these reports, surgical procedures encompassed bronchial reconstruction, pulmonary artery reconstruction, and even total lung resection, all of which are highly invasive interventions. The median operating time of 272 min (range 126–513) and intraoperative blood loss of 270 ml (range 50–4000) closely aligns with findings from surgery following CRT [12], and the presence of a few cases with blood loss exceeding 1000 ml underscores the complexity of these procedures. Furthermore, grade ≥ 3 postoperative complications and instances of bronchial stump fistula formation were observed. In the present case, severe adhesions and fibrosis were observed in the lung apex, presumably because of the effects of preoperative treatment. This necessitated combined resection of the chest wall.

**Table 1:** Previously reported cases of salvage surgery for locoregional recurrence following the PACIFIC regimen

Author	Age/sex	cTNM	Histology	RT-dose (Gy)	Cycles durva	ycTNM	Type of resection	Operating time (mins)	Perioperative complication	Blood loss (ml)	90-day post-op complications (Clavien-Dindo Gr III-V)	Reintervention < 30 days	pTNM	Margin	Ef	Survival (months)
Dickhoff C. [3]	53/F	TxN2	Large	66	25	T2aN0	LUL	159	–	50	–	–	T1cN1	R0	ND	9 (dead)
	53/M	T4N0	Ad	66	19	T2N0	RUL	525	Tear azygos v./PA	1100	–	–	T1bN0	R0	ND	23 (alive)
	64/M	T4N2	SCC	60	18	T1cN0	LLL	126	–	75	–	–	T2aN0	R0	ND	23 (alive)
	56/M	T4N1	SCC	65	24	T1bN2	RUL	237	–	100	–	–	T2aN2	R0	ND	19 (alive)
	56/M	T2N2	SCC	66	25	T1bN0	LP	444	Tear PA	4000	BPF, sepsis, bleeding	Thoracotomy x2	T2bN0	R0	ND	9 (dead)
	57/M	T3N2	SCC	60	9	T2aN0	RP	245	–	500	–	–	T2bN1	R0	ND	5 (alive)
	59/F	T2bN3	Ad	60	4	T3N0	LUL (sleeve PA)	213	–	300	–	–	T3N0	R1	ND	5 (alive)
	73/F	T1N2	SCC	60	3	T2aN0	RUL	232	–	50	–	–	T3N0	R0	ND	4 (alive)
	67/M	T2N2	SCC	60	10	T3N0	RLL	298	–	150	–	–	T3N2	R0	ND	2 (alive)
	74/M	T4N0	SCC	55	12	T3N0	RML/RLL	358	–	400	–	–	T4N0	R0	ND	1 (alive)
Minegishi K. [5]	61/M	T4N2	SCC	66	24	T2aN1	RUL (PA/Br plasty)	272	–	800	–	–	T2aN0	R0	ND	10 (alive)
Ueno T. [10]	76/M	T1cN2	SCC	60	ND	T3N0	RUL	195	–	70	–	–	T3N0	R0	1b	18 (alive)
	54/M	T3N2	SCC	60	ND	T3N0	RLL	513	–	1330	–	–	T1bN0	R0	1b	4 (alive)
Funaki S. [11]	70/M	T4N0	SCC	60	24	ND	RUL+CW(2-3th)	ND	–	ND	–	–	T1aN0	R0	2	24 (alive)
Takenaka M. [2]	72/F	T3N1	SCC	66	ND	T3N0	RUL+CW(1-4th)	273	–	100	–	–	T3N0	R0	2	17 (alive)
Our case	53/M	T4N3	Ad	60	24	T3N0	LUL+CW(1-4th)	389	PA embolism	270	–	–	T0N0	R0	3	7 (alive)

*M* Male, *F* Female, *cTNM* Clinical TNM stage of index tumor, *pTNM* Pathological TNM stage, *Large* Large cell carcinoma, *Ad* Adenocarcinoma, *SCC* Squamous cell carcinoma, *RT* Radiotherapy, *LUL* Left upper lobectomy, *RUL* Right upper lobectomy, *LLL* Left lower lobectomy, *LP* Left pneumonectomy, *RP* Right pneumonectomy, *PA* Pulmonary artery, *Br* Bronchus, *CW* Chest wall resection, *ND* No data, *R0* Radical resection, *R1* Microscopic non-radical resection

In addition, the present case represents the second documented case study of a superior sulcus tumor (SST) that underwent salvage surgery after treatment with the PACIFIC regimen [8]. It is the first documented case of this tumor type in the context of contralateral mediastinal and supraclavicular lymph node metastasis (cN3), which was considered oncologically unresectable at the time of the diagnosis. The uniqueness of this case and its successful outcome highlight the feasibility of salvage surgery after the PACIFIC regimen for such patients and emphasize the importance of individualized treatment strategies.

**Fig. 5:**
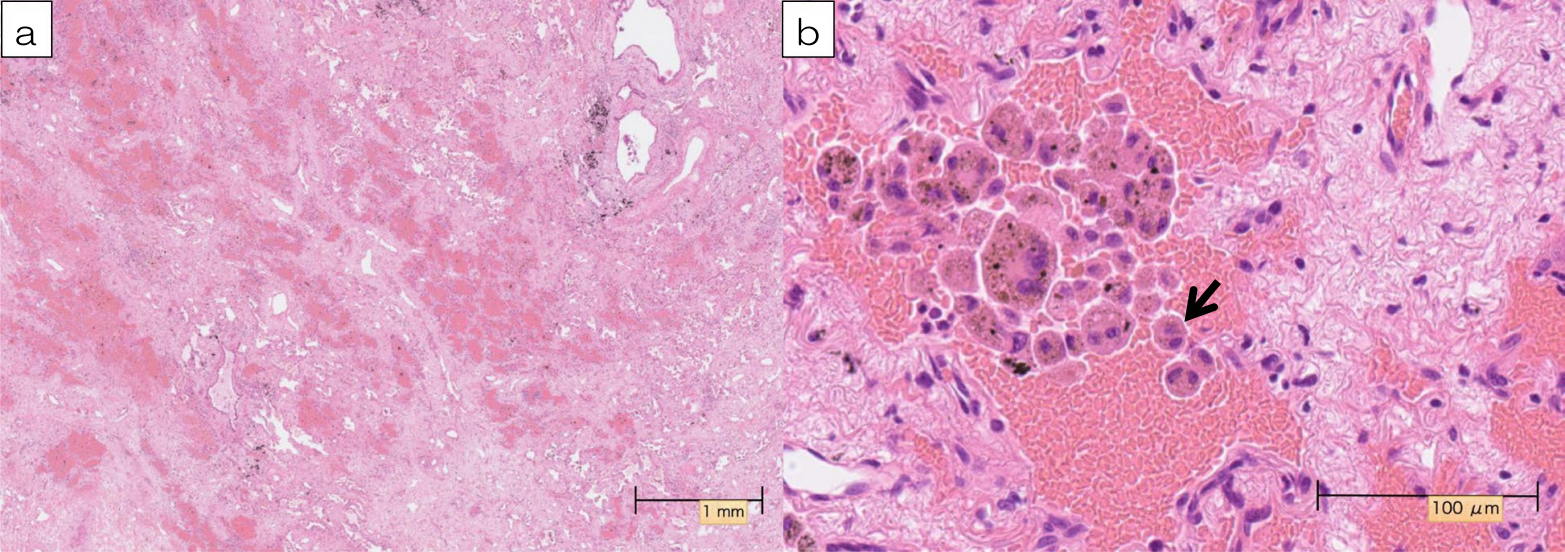
**a** Microscopically, hematoxylin, and eosin staining of the tumor showed no viable tumor cells in any surgical specimen. Extensive fibrosis with chronic inflammation, hemosiderin deposition, and bleeding were observed at the site where FDG accumulation was originally observed (magnification × 1.25). **b** The infiltration of atypical multinucleated giant cells (arrow) was also observed (magnification × 40)

There are concerns about the accuracy of the diagnosis before surgery. In the present case, CT and FDG-PET before surgery showed both enlargement and residual FDG uptake in the primary lesion, which was later explained by histopathological findings of hemosiderin deposition and giant cell infiltration (Fig. 5). This led us to speculate that the enlargement and the residual uptake were reflective of bleeding and associated inflammation. In the context of immunotherapy, it is important to consider that transient enlargement of the tumor burden, caused by bleeding and infiltration of inflammatory cells, may occur either in a delayed manner or repeatedly throughout the disease course [13, 14]. Some reports have shown the utility of modalities such as liquid biopsy with cell-free DNA (cfDNA) and circulating tumor DNA (ctDNA), as well as imaging examinations, such as PET and magnetic resonance imaging, for assessing the activity and metastatic potential of tumors in patients with NSCLC. The accurate evaluation of minimal residual disease after the treatment of lung cancer is still in the early stages of development [15, 16], and such technologies should be investigated in the near future.

## Conclusions

Salvage surgery after the PACIFIC regimen for initially unresectable NSCLC with N3 involvement may be feasible in selected patients, offering a potential treatment option to control local disease. Long-term follow-up should be conducted to evaluate the effectiveness of salvage surgery.

## Data Availability

Not applicable.

## Abbreviations

NSCLC: Non-small cell lung cancer
SST: Superior sulcus tumor
CRT: Chemoradiotherapy
CT: Computed tomography
CTCAE: Common terminology criteria for adverse events
FDG: Fluorodeoxyglucose
FDG-PET: 18F-fluorodeoxyglucose-positron emission tomography
PD-L1: Programmed death ligand 1
OS: Overall survival
PFS: Progression-free survival
SST: Superior sulcus tumor
cfDNA: Cell-free DNA
ctDNA: Circulating tumor DNA
MRI: Magnetic resonance imaging
MRD: Minimal residual disease
